# A novel mutation in the *CRYAA* gene associated with congenital cataract and microphthalmia in a Chinese family

**DOI:** 10.1186/s12881-018-0695-5

**Published:** 2018-10-19

**Authors:** Zixun Song, Nuo Si, Wei Xiao

**Affiliations:** 1 Department of Ophthalmology, Shengjing Hospital, China Medical University, Shenyang, 110004 Liaoning China; 2 McKusick-Zhang Center for Genetic Medicine, Institute of Basic Medical Sciences Chinese Academy of Medical Sciences, School of Basic Medicine Peking Union Medical College, Beijing, 100005 China

**Keywords:** Congenital cataracts, Mutation, *CRYAA* gene

## Abstract

**Background:**

Congenital cataract is the leading cause of blindness in children worldwide. Approximately half of all congenital cataracts have a genetic basis. Protein aggregation is the single most important factor in cataract formation.

**Methods:**

A four-generation Chinese family diagnosed with autosomal dominant congenital cataracts and microphthalmia was recruited at the Shengjing Hospital of China Medical University. Genomic DNA was extracted from the peripheral blood of the participants. All coding exons and flanking regions of seven candidate genes (*CRYAA, CRYBA4, CRYBB2, CRYGC, GJA8, MAF,* and *PITX3*) were amplified and sequenced. Restriction fragment length polymorphism (RFLP) assays were performed to confirm the candidate causative variant, c.35G > T in the *CRYAA* gene. We constructed pcDNA3.1(+)-CRYAA expression plasmids containing either the wild-type or the R12L mutant alleles and respectively transfected them into HEK293T cells and into HeLa cells. Western blotting was performed to determine protein expression levels and protein solubility. Immunofluorescence was performed to determine protein sub-cellular localization.

**Results:**

A heterozygous variant c.35G > T was identified in exon 1 of *CRYAA*, which resulted in a substitution of arginine to leucine at codon 12 (p.R12L). The nucleotide substitution c.35G > T was co-segregated with the disease phenotype in the family. The mutant R12L-CRYAA in HEK293T cells showed a significant increase in the expression level of the CRYAA protein compared with the wild-type cells. Moreover, a large amount of the mutant protein aggregated in the precipitate where the wild-type protein was not detected. Immunofluorescence studies showed that the overexpressed mutant CRYAA in HeLa cells formed large cytoplasmic aggregates and aggresomes.

**Conclusions:**

In summary, we described a case of human congenital cataract and microphthalmia caused by a novel mutation in the *CRYAA* gene, which substituted an arginine at position 12 in the N-terminal region of αA-crystallin. The molecular mechanisms that underlie the pathogenesis of human congenital cataract may be characterized by the prominent effects of the p.R12L mutation on αA-crystallin aggregation and solubility. Our study also expands the spectrum of known *CRYAA* mutations.

## Background

Congenital cataract is the leading cause of blindness in children worldwide [1]. Cataracts are characterized by an opacification of the eye’s lens presenting at birth or shortly thereafter. More than half of the cases of congenital cataracts are associated with other ocular anomalies, such as nystagmus, strabismus, microcornea or microphthalmia, persistent hyperplastic primary vitreous, congenital glaucoma, morning glory syndrome, and persistent pupillary membrane [2]. The prevalence of congenital cataracts is 1 to 6 per 10,000 live births [3]. Approximately 50% of congenital cataracts have a genetic basis [4]. Mutations in a number of genes have been reported to be associated with various types of congenital cataracts. Most of the mutations occur in genes encoding crystallins, membrane proteins, and filament proteins.

Crystallin proteins constitute up to 90% of the soluble proteins in the lens and are composed of three groups, α-, β- and γ- crystallins [5]. α-crystallin belongs to the small heat shock protein (sHSP) family, preventing aggregation of partially unfolded proteins [6]. The α-crystallin protein family consists of two similar proteins, αA-crystallin, encoded by the *CRYAA* gene, and αB-crystallin, encoded by *CRYAB* [7]. So far, there have been a total of 26 mutations identified in the *CRYAA* gene. Previous studies have shown that mutations in the *CRYAA* gene were responsible for congenital cataracts, either alone or in association with other pathological conditions such as microcornea or microphthalmia [8]. This revealed that αA-crystallin plays a major role in the development of the lens.

This study involves a four-generation Chinese family with congenital cataracts and microphthalmia. By directly screening the candidate genes, we found a novel αA-crystallin (CRYAA) mutation (p.R12L). The effects of this mutation on the αA-crystallin protein were further studied. We cloned the mutant protein and transiently expressed it in cell lines. The mutant CRYAA protein exhibited reduced solubility, with related consequent protein aggregation and cataract formation. To our knowledge, the mutation has not been previously reported.

## Methods

### Subjects

A four-generation Chinese family diagnosed with autosomal dominant congenital cataract (ADCC) and microphthalmia was recruited at the Shengjing Hospital of China Medical University (Liaoning, China). To confirm the affected status and identify whether there were any other ocular abnormalities, all the family members that participated in the study underwent complete ophthalmic examinations, including visual acuity, slit-lamp biomicroscopic fundus examination with dilated pupils, axial length, and B-scan ultrasonography. The phenotype of the proband was documented by ophthalmic operating microscope photography. A total of 100 unrelated subjects with no family history of congenital cataracts were also recruited as controls. Written informed consent was obtained from all the participants enrolled in our study or from their guardians. This study was performed in accord with the tenets of the Declaration of Helsinki and was approved by the Ethics Committee of Shengjing Hospital.

### DNA analysis and gene sequencing

Genomic DNA of the participants was extracted from peripheral blood using the QIAamp DNA Blood Mini Kit (Qiagen; Valencia, CA, USA). All coding exons and flanking regions of seven known ADCC genes (accompanied by microphthalmia phenotype) *CRYAA, CRYBA4, CRYBB2, CRYGC, GJA8, MAF,* and *PITX3* were amplified by genomic polymerase chain reaction (PCR). These functional candidate genes were determined from the Online Mendelian Inheritance in Man (OMIM) database. The primer sequences and cycling conditions used in the PCR were obtained from earlier reports [9]. The PCR products were sequenced. The sequences obtained from the proband and one unaffected family member were aligned to a human reference sequence (hg19).

### Causative variants identification and validation

The candidate causative variant, c.35G > T in the *CRYAA* gene, created a *Sac*I restriction site. A restriction fragment length polymorphism (RFLP) assay was performed to detect the variant. To confirm the mutation, DNA sequences from all available family members and 200 unrelated individuals were amplified by PCR using the primers: 5’-CATCCAGCACCCCTGGTTCGAG-3′ (forward) and 5’-CTTACCTCAGAGATGCCGGAGT-3′ (reverse).

PCR fragments were digested with *Sac*I (New England Biolabs; Ipswich, MA, USA) and separated by 8% polyacrylamide gel electrophoresis. The American College of Medical Genetics guidelines were used to interpret variant pathogenicity. Combined Annotation-Dependent Depletion (CADD), Sorting Intolerant from Tolerant (SIFT) and PolyPhen2 algorithms were used to predict the possible functional impact of the amino acid change.

### Plasmid construction

We constructed wild-type and R12L mutant CRYAA expression plasmids. Wild-type sequences of full-length alpha-crystallin A chain (NM_000394) coding regions were amplified from human thymus first-strand cDNA (Human multiple tissues cDNA panels; BD Biosciences; Palo Alto, CA, USA) and were cloned into the pcDNA3.1(+) vector (Invitrogen, CA, USA) at the multiple cloning site of *Nhe*I and *EcoR*I. The primers used for PCR amplification were 5′- CTAGCTAGCGCCACCATGGACGTGACCATCCAGCAC-3′ (forward) and 5′- CCGGAATTCTTAGGACGAGGGAGCCGAGGT-3′ (reverse). To generate the mutant (c.35G > T; p.R12L) alpha-crystallin A, site-directed mutagenesis was carried out with the following oligonucleotide primer and its complement: sense primer, 5’-ACCCCTGGTTCAAGCTCACCCTGGGGCCCTT-3′; antisense primer, 5’-AAGGGCCCCAGGGTGAGCTTGAACCAGGGGT-3′. All constructs were directly sequenced to confirm the inserted fragments.

### Cell culture and transfection

Human embryonic kidney 293 T (HEK293T) cells and HeLa cells were grown in Dulbecco’s modified Eagle medium (DMEM) with high glucose supplemented with 10% foetal bovine serum (FBS) at 37 °C with 5% CO2. The cells for transfection were cultured on six-well plates and grown till 70–90% confluent. Wild-type and R12L mutant pcDNA3.1-CRYAA plasmids were separately transfected into the cells in comparable amounts using Lipofectamine 3000 (Invitrogen Corporation, Carlsbad, CA, USA).

### Protein expression analysis

Twenty-four hours after transfection, the cultured HEK293T cells were harvested and analysed by Western blotting with an antibody to αA crystallin. HEK293T cells were lysed in RIPAII lysis buffer (Beyotime, Shanghai, China) containing a protease inhibitor cocktail (Roche, Basel, Switzerland) for 10 min at 4 °C. The samples were centrifuged at 12,000 g for 10 min at 4 °C. The supernatant was collected; the precipitates containing insoluble proteins were washed three times with ice cold PBS and were conducted with 6 mol/L urea. Protein concentration was determined by the bicinchoninic acid (BCA) protein assay (Thermo Scientific-Pierce Chemical Co, Rockford IL). Twenty micrograms of extracts was denatured in SDS loading buffer (TaKaRa), loaded on the gel, separated by 15% SDS-PAGE gel electrophoresis and transferred to PVDF membranes (Millipore, Massachusetts, USA). The membranes were blocked and incubated with anti-alpha A crystallin antibody (Abcam) and anti-β-actin IgG (Sigma-Aldrich, Saint Louis, USA) at a 1:1000 dilution at 4 °C overnight, followed by HRP-conjugated goat anti-rabbit IgG (Pierce, Rockford, USA) and HRP-conjugated goat anti-mouse IgG (Pierce, Rockford, USA) at a 1:3000 dilution for one hour at room temperature. Enhanced chemiluminescence (ECL) reagents (SuperSignal West Femto Maximum Sensitivity Substrate, Thermo Fisher Scientific, Rockford, USA) were used and the signal was detected. The intensity of the αA crystallin bands was normalized relative to β-actin bands and was analysed by ImageJ software. The expression level of αA crystallin was calculated as the mean of three independent experiments.

### Immunofluorescence for aggresome detection

HeLa cells were cultured on glass bottom dishes. Twenty-four hours after transfection, the cells were fixed with 4% paraformaldehyde for 30 min permeabilized with 0.05% Triton-X 100 for 10 min and blocked with 5% bovine serum albumin (BSA) for 1 h. The cells were incubated with anti-alpha A crystallin antibody (Abcam) at a 1:200 dilution at 4 °C overnight, followed by Dylight 488 goat anti-rabbit IgG (Earthox, San Francisco, CA, USA) at a 1:800 dilution for one hour at room temperature. The nucleus was stained with 500 ng/ml of DAPI (Sigma Aldrich). The images were acquired with Olympus IX81 confocal fluorescence microscopy and analysed by FV1000 Viewer (Ver.3.0a) software (Olympus). The assays were repeated three times.

## Results

### Clinical features

**Fig. 1:**
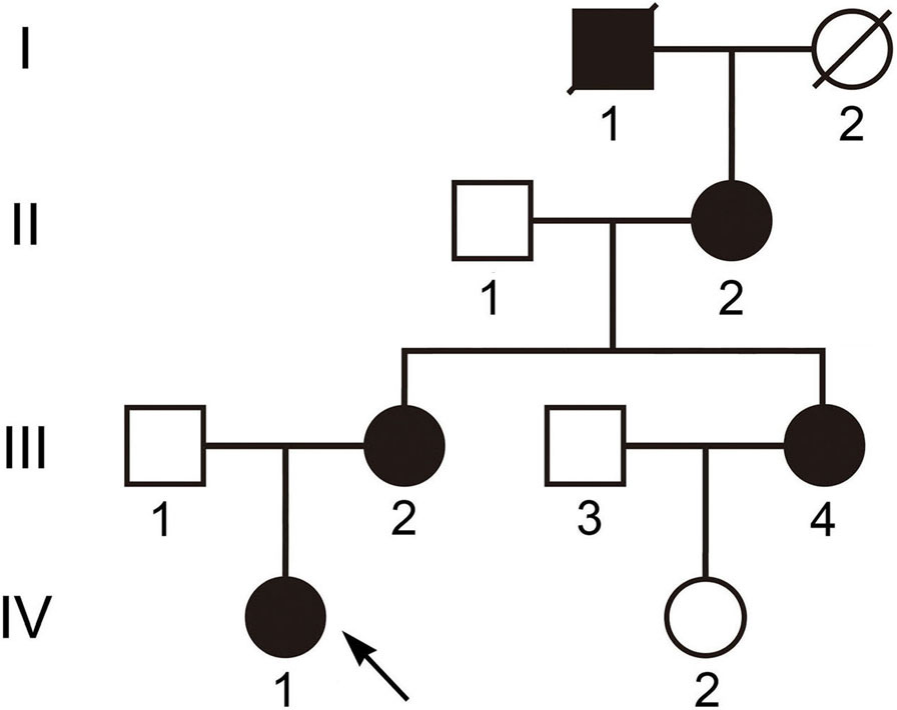
Pedigree of the Chinese family with autosomal dominant congenital cataracts and microphthalmia. Squares and circles indicate males and females, respectively. Filled symbols indicate affected members, and empty symbols indicate unaffected individuals. The diagonal line indicates a deceased family member and the arrow indicates the proband

**Fig. 2:**
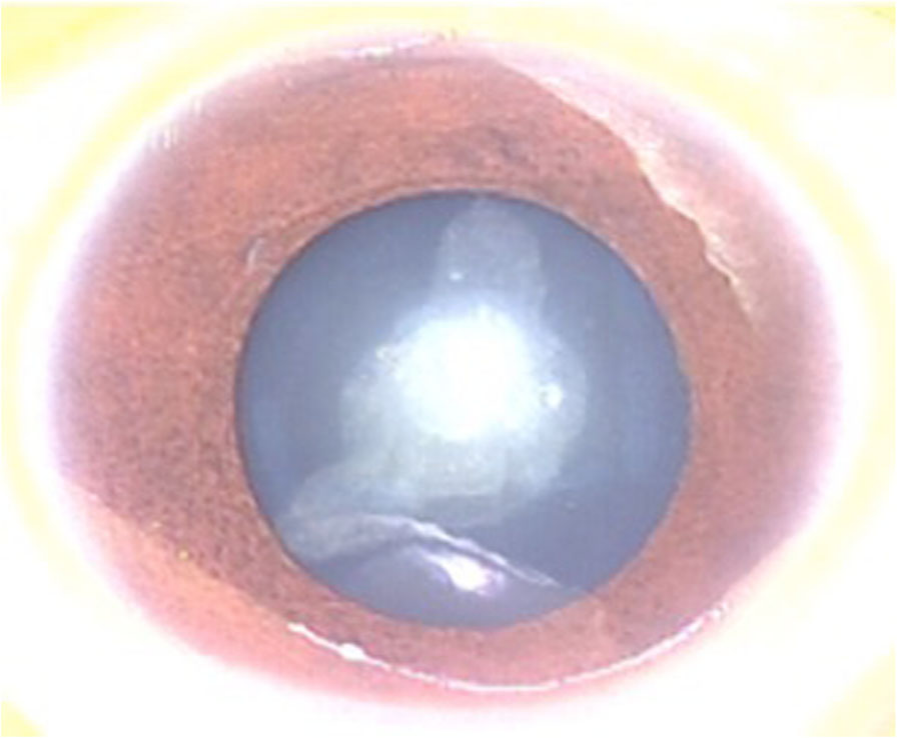
Photograph of the right eye of the proband. The phenotype of the proband (IV: 1) was documented by ophthalmic operating microscope photography before surgery and shows a nuclear cataract and microphthalmia. The same phenotype was noted bilaterally

Members of a four-generation Chinese family affected with ADCC and microphthalmia were analysed from clinical and genetic perspectives (Fig. 1). The proband, a 10-year-old girl, was diagnosed as having bilateral nuclear congenital cataracts and microphthalmia after birth (Fig. 2). The girl underwent surgery for cataract aspiration shortly afterwards. The eyeballs of the girl were too small to implant intraocular lenses (IOL). The axial length was only 19.37 mm and 19.48 mm at last follow up. The refractive error was corrected with hyperopia frame glasses and the best-corrected visual acuity of the proband was 0.4. The affected individuals of the family showed similar phenotypes. No systemic disease or other ocular disease was found. Dominant inheritance was supported by the presence of affected individuals in each generation.

### A *CRYAA* missense mutation

**Fig. 3:**
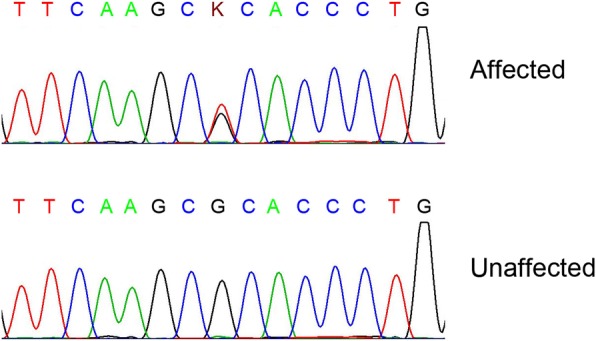
A novel missense mutation (c.35G > T; p.R12L) in *CRYAA* in a Chinese family with cataracts and microphthalmia. DNA sequences of *CRYAA* in affected and unaffected individuals. A heterozygous variant c.35G > T was identified in exon 1 of *CRYAA*. The upper chromatogram of the DNA sequence from the affected proband (IV: 1) shows both G and T (K) at position 35; thus, the nucleotide variant resulted in the substitution of an arginine for a leucine at codon 12 (p.R12L). The lower sequence chromatogram from an unaffected individual (II: 1) shows only the wild-type CRYAA allele

**Fig. 4:**
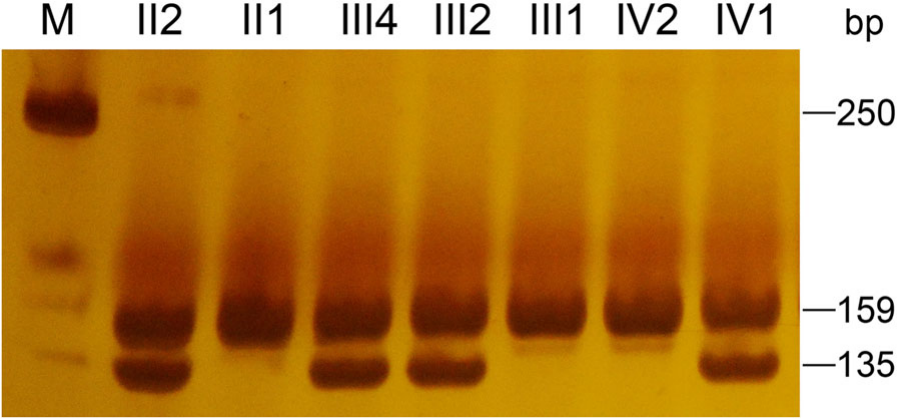
Polyacrylamide gel electrophoresis confirmed that the nucleotide substitution c.35G > T co-segregated with the disease phenotype in the family. The variant was observed in the proband and her affected mother, aunt, and grandmother, but not in her unaffected father, sister, grandfather or 100 unrelated Chinese controls. The PCR product was 159 bp in length and contained a *Sac*1 site. The mutant allele contains a *Sac*1 restriction site and can be digested into two fragments (135 bp, 24 bp) by *Sac*1, but the wild-type allele cannot be digested. Affected individuals show two bands (135 bp and an undigested band of 159 bp) on an 8% polyacrylamide gel. All the unaffected members show only a single undigested band of 159 bp

Seven candidate genes were directly sequenced. A heterozygous variant c.35G > T was identified in exon 1 of *CRYAA* (Fig. 3). The nucleotide variant resulted in an arginine to leucine substitution at codon 12 (p.Arg12Leu). Polyacrylamide gel electrophoresis confirmed that the nucleotide substitution c.35G > T was co-segregated with the disease phenotype in the family (Fig. 4). The variant was observed in the proband and her affected mother, aunt, and grandmother, but not in her unaffected father, sister, grandfather or in 200 unrelated Chinese controls. As assessed by the ACMG/AMP 2015 guideline, the p.Arg12Leu alteration in the CRYAA protein would be classified as “likely pathogenic”. The functional impact of the amino acid change was predicted damaging based on multiple algorithms (predicted to affect protein function by SIFT, rated as probably damaging by PolyPhen-2, and a CADD score of 27.2).

### The mutant CRYAA has an increased protein expression level and decreased solubility

**Fig. 5:**
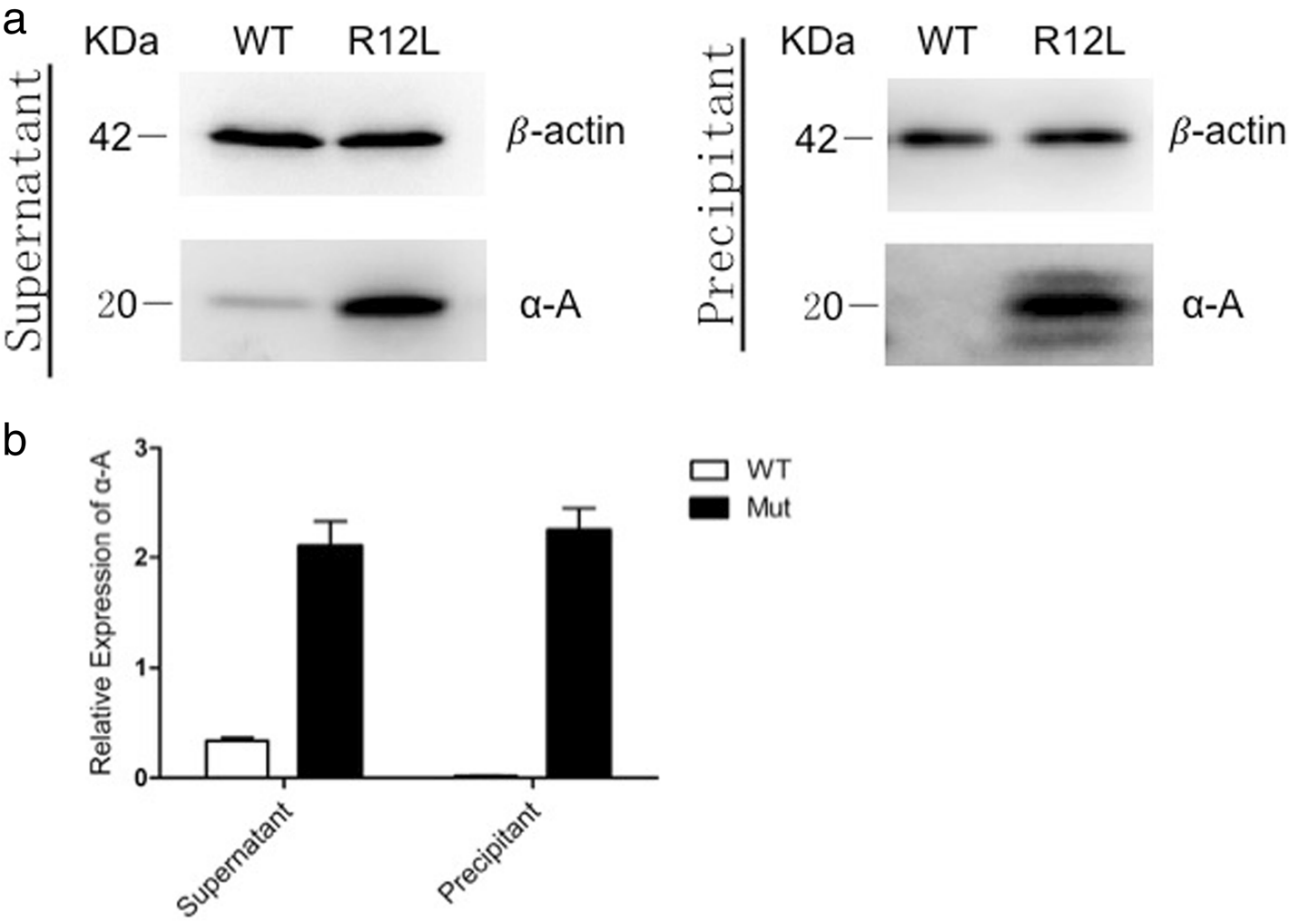
Protein expression levels of WT-CRYAA and R12L-CRYAA transfected into HEK293T cells. Western blots were performed with the anti-αA crystallin antibody. β-actin was used as the loading control. (**a**) The mutant R12L-CRYAA in HEK293T cells had a greatly increased level of protein expression when compared with the wild-type allele. Moreover, a large amount of the mutant protein aggregated in the precipitate where the wild-type protein was not detected. Expression levels of the β-actin loading control in both wild-type and the mutant proteins were comparable. (**b**) Each bar represents the mean ± standard deviation of three individual experiments. The difference was significance (*p* < 0.01)

To assess the protein expression level and the protein properties, untagged pCDNA3.1 with either wild-type or the mutant R12L-CRYAA constructs were transiently expressed in HEK293T cells and were detected by immunoblot analysis using an anti-αA-crystallin antibody. In transfected cells, a 20-kDa band was detected, which agreed with the size of the full length αA-crystallin protein (Fig. 5). The mutant R12L-CRYAA showed a significant increase in the level of protein expression compared with the wild-type protein. Moreover, a large amount of the mutant protein aggregated in the precipitate where the wild-type protein was not detected. Expression levels of the β-actin loading control in the lanes of both the wild-type and mutant samples were comparable. These results indicate that the R12L mutation increased the protein level and decreased the solubility of the αA-crystallin.

### Mutant αA-crystallins in HeLa cells form aggresomes

**Fig. 6:**
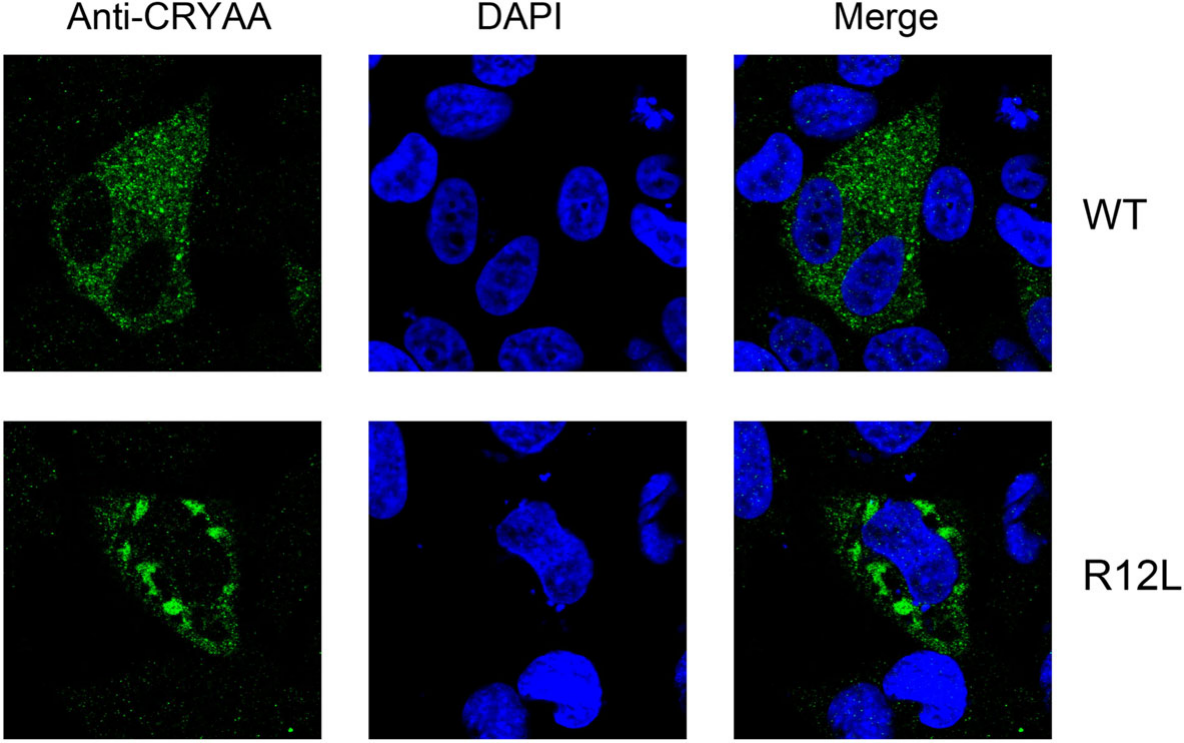
Localization of wild-type and R12L mutated αA-crystallin proteins. Photomicrographs show the distribution of immunoreactive αA-crystallin (green) and DAPI-stained nuclei (blue) in HeLa cells. Both wild-type CRYAA and mutated R12L-CRYAA proteins were predominantly localized in the cytoplasm. Cells transfected with wild-type CRYAA showed a homogenous distribution of the protein in the cytoplasm and there was little or no aggregation in the cells. In contrast, the cells transfected with the mutant R12L-CRYAA showed significant intracellular aggregation in a number of cells

To further show the subcellular localization of wild-type CRYAA or mutated R12L-CRYAA in transfected HeLa cells, the cells were subjected to immunofluorescence assays with an anti-alpha A crystallin antibody. Photomicrographs show the distribution of immunoreactive αA-crystallin (green) and DAPI-stained nuclei (blue) in the HeLa cells (Fig. 6). Both wild-type CRYAA and mutated R12L-CRYAA were predominantly localized in the cytoplasm. Cells transfected with wild-type CRYAA showed a homogenous distribution of its expression in the cytoplasm and there was a little or no aggregation in the cells. In contrast, significant intracellular aggregation was seen in a number of the cells transfected with the mutant R12L-CRYAA.

## Discussion

In the present study, we describe a Chinese family suffering from congenital cataracts and microphthalmia. As the size of the family was not large enough for linkage analysis, we analysed functional candidate genes. Using direct sequencing, we identified a novel missense mutation (c.35G > T; p.R12L) in CRYAA/αA-crystallin. To date, there are a total of 26 other mutations that have been identified in CRYAA [8], including 21 missense mutations, 1 regulatory mutation, and 4 small deletions. To our knowledge, the mutation has not been reported earlier although another mutation of the same residue, R12C, is a highly recurrent mutation that has been reported in various studies. The R12C mutation was first reported by Lars Hansen [10] in a family afflicted with cataracts and microcornea in 2007. The phenotype of the R12C mutation in the reported cases is similar to that in the present study. Li-Yun Zhang [11] further showed that the R12C mutation altered the heat-shock response, which suggested a change of chaperoning capacity and networking.

Crystallins are the major structural proteins in the lens accounting for up to 90% of total soluble proteins [12]. The crystallin superfamily is composed α-, β- and γ-crystallins which contribute to the maintenance of lens transparency and to a proper refractive index of the lens [13]. Crystallins are some of the most highly concentrated and most highly soluble intracellular proteins in the human body. αA-crystallin is a member of the small heat-shock-protein (sHSP) family, which has chaperone-like activity [14]. It delays aggregation of various proteins under a wide range of stress conditions through interactions with non-native and aggregate-prone protein states [15]. Previous studies have also shown that alpha crystallins may play a possible role in stimulating epithelial cell differentiation in the lens [16]. Furthermore, a knockout mouse strain with an αA crystallin gene deletion shows microphthalmia and eventual opacity of the lens, which demonstrates the important role of alpha A crystallin in the development and maintenance of lens transparency [17].

**Fig. 7:**
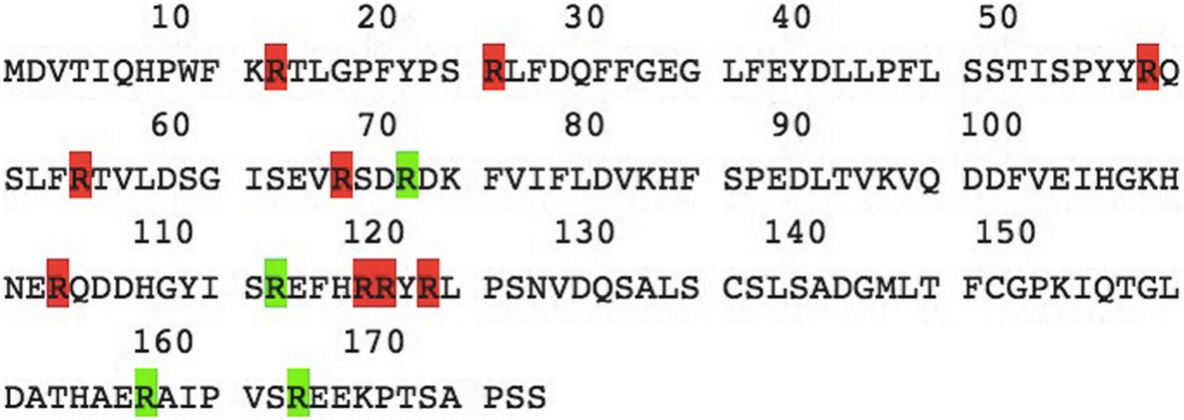
Protein sequence of αA-crystallin. There are 13 arginine residues (red or green) in human αA-crystallin. Nine of these 13 arginine residues (red) are hot spots for mutations, which eventually lead to congenital cataracts

In addition to the R12L mutation, there are a total of 26 other mutations identified in CRYAA [8]. Interestingly, when we carefully investigated the 26 mutations associated with congenital cataracts in αA-crystallin, we observed that the frequency of arginine mutations made up more than half of the total. Arginine residues are highly conserved in αA-crystallin in all mammalian species. There are 13 arginine residues in human αA-crystallin. Nine of these 13 arginine residues are hot spots for mutations which eventually lead to congenital cataracts (Fig. 7). In addition, the post-translational modification of several arginine residues contributes significantly to the chemical modification of lens proteins in ageing and cataractogenesis [18]. All these facts suggest that arginine residue may play a crucial role in the structure, stability, and molecular chaperone function of αA-crystallin [19].

αA-crystallin contains a hydrophobic N-terminal region (NTR), a highly conserved αA-crystallin domain (ACD) and a short hydrophilic C-terminal [20, 21]. The R12L mutation is located in the NTR of αA-crystallin. The NTR remains the most enigmatic region as its properties are difficult to study biochemically and structurally [22]. Nevertheless, it is clear that the NTR is essential for the assembly of oligomers, maintaining the polydisperse nature of α-crystallin, and retaining its high solubility [23–25]. Previous studies have shown that N-terminally truncated crystallins destabilize the formation of large oligomeric species [25]. Insertion of a small portion (14 residues) of the NTR of HSPB1/HSP27 into that of a bacterial sHSP that forms distinct 24-mers that can be crystallized generated a chimeric sHSP that forms a 48-mer [26]. All these findings imply that the formation of oligomers is NTR context dependent. One factor that may contribute to the aggregation and insolubility of the R12L mutant is that the substitution of an arginine for a leucine residue occurs in the N-terminal region of α-crystallin.

Our expression studies demonstrated that the R12L mutation increased the protein level and decreased the solubility of the αA-crystallin, which forms aggresomes in the cytoplasm. Protein aggregation is the single most important factor in cataract formation [27]. Protein aggregation problems have been observed extensively in many types of crystallin proteins, such as αA (R116C) [7], αB (R120G) [28], βB2 (V187E) [29], γC (G129C) [30], and γD (W43R) [31], which are known to cause congenital cataracts. Furthermore, Zhao L [32] reported that when the protein aggregation caused by mutant crystallin proteins was decreased by lanosterol treatment, the lens clarity in animal models was increased and the cataract severity was reduced. It is apparent that protein aggregation contributes to the pathogenesis of ADCC, although the precise mechanisms by which lens protein aggregation causes opacification are not completely understood.

## Conclusions

In conclusion, we described a case of human congenital cataracts and microphthalmia caused by a novel mutation in the *CRYAA* gene, which substituted an arginine at position 12 in the N-terminal region of αA-crystallin. The molecular mechanisms that underlie the pathogenesis of human congenital cataracts may be characterized by the prominent effects of the p.R12L mutation on αA-crystallin aggregation and solubility. Our study also expands the spectrum of known *CRYAA* mutations.

## Abbreviations

ADCC: autosomal dominant congenital cataract
BSA: bovine serum albumin
CADD: Combined Annotation-Dependent Depletion
DAPI: 4′,6′-diamidino-2-phenylindole
ECL: Enhanced chemiluminescence
FBS: foetal bovine serum
HEK293T cell: human embryonic kidney 293 T cells
IOL: intraocular lens
NTR: N-terminal region
OMIM: Online Mendelian Inheritance in Man database
PCR: polymerase chain reaction
RFLP: restriction fragment length polymorphism
sHSP: the small heat-shock-protein
SIFT: Sorting Intolerant from Tolerant
